# Supplementary material for: Effect of crystal facets in plasmonic catalysis
Source: Nat Commun. 2024 May 9;15:3923. doi: 10.1038/s41467-024-47994-y (PMC11519563; doi:10.1038/s41467-024-47994-y)
Supplement: Supplementary file 1 — Supplementary Information [file 41467_2024_47994_MOESM1_ESM.pdf]

*Supporting information*

# Effect of Crystal Facets in Plasmonic Catalysis

*Yicui Kang [1&], Simão M João [2 &], Rui Lin[1*], Kang Liu [3], Li Zhu[1], Junwei Fu [3], Weng-Chon (Max) Cheong[4], Seunghoon Lee[1,5], Kilian Frank[6], Bert Nickel[6], Min Liu[3], Johannes Lischner[2*], Emiliano Cortés[1*]*

[1] Nanoinstitute Munich, Faculty of Physics, Ludwig-Maximilians-Universität München, 80539 München, Germany

[2] Departments of Materials and Physics and the Thomas Young Centre for Theory and Simulation of Materials, Imperial College London, London, U.K.

[3] Hunan Joint International Research Center for Carbon Dioxide Resource Utilization, School of Physics and Electronics, Central South University, Changsha 410083, P.R. China

[4]Faculty of Innovation Engineering (FIE), Macau University of Science and Technology, 999078, Macau, P.R. China

[5] Department of Chemistry, Dong-A University, Busan, 49315 South Korea

[6] Faculty of Physics and Center for Nanoscience (CeNS), Ludwig-Maximilians-Universität, Geschwister-Scholl-Platz 1, 80539 München, Germany

[&]Contributed equally

*E-mail: Rui.Lin@physik.uni-muenchen.de, j.lischner@imperial.ac.uk, Emiliano.Cortes@lmu.de

**Figures**

Figure S1. TEM images of Au NPs.

Figure S2. Size histogram of Au NPs.

Figure S3. TEM images of Au/C composite.

Figure S4. ECSA measurement result.

Figure S5. I-t curves of Au NPs in the $CO_2$RR system.

Figure S6. LSV curves of Au NPs in the $CO_2$RR system.

Figure S7. $^1$H NMR spectrum.

Figure S8. Control experiment of carbon in the $CO_2$RR system.

Figure S9. Partial current density of Au NPs in the electrocatalytic $CO_2$RR system.

Figure S10. Model of DFT calculation and free energy diagrams in the $CO_2$RR system.

Figure S11. $CO_2$RR performance under 405 nm illumination.

Figure S12. LSV curves of Au NPs and the free energy diagrams in the HER system.

Figure S13. Stability measurement of Au NPs in the electrocatalytic $CO_2$RR system.

Figure S14. Control experiment under Ar bubbling.

Figure S15. Experimental results of HER system.

Figure S16. Mechanism investigation with 405 nm illumination.

Figure S17. $CO_2$RR measurement under high temperature.

Figure S18. Setup scheme of $CO_2$RR measurement.

Figure S19. Setup scheme of HER measurement.

**Tables**

Table S1. ICDD XRD Standard card PDF#04-0784.

Table S2. Elemental analysis (C, H, N) (EA) test of Au/C.

Table S3. The ECSA values of working electrodes.

Table S4. Edge/facet ratio of Au NPs.

Table S5. Population of hot carriers of intra- and interband transitions.

## SUPPLEMENTARY FIGURES

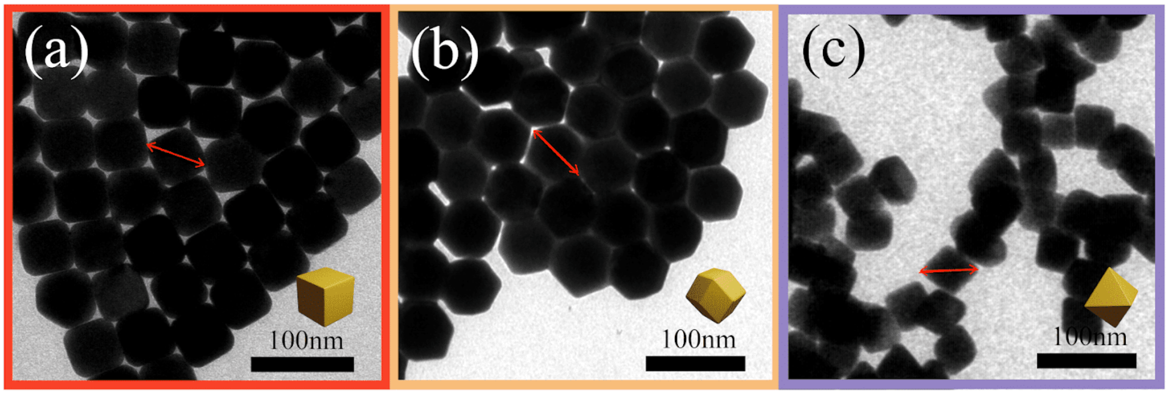


**Figure S1.** TEM images of (a) Au NCs (b) Au RDs and (c) Au OCs. The red arrow indicators within the figure delineate the dimensions that were measured for size distribution shown in Figure S2.

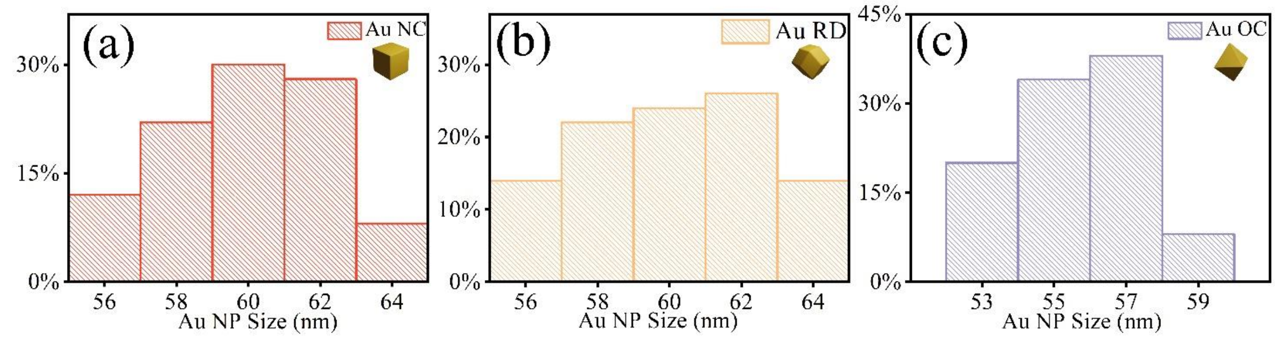


**Figure S2.** Size distribution of (a) Au NCs (b) Au RDs and (c) Au OCs. The dimensions that were measured were delineated by the red arrow indicators in Figure S1. By conducting 100 measurements on Au NPs from distinct synthesizing batches, average sizes of NC 60nm, RD 60nm and OC 56nm were ascertained. All the relevant Source data in SI are provided as a Source Data file.

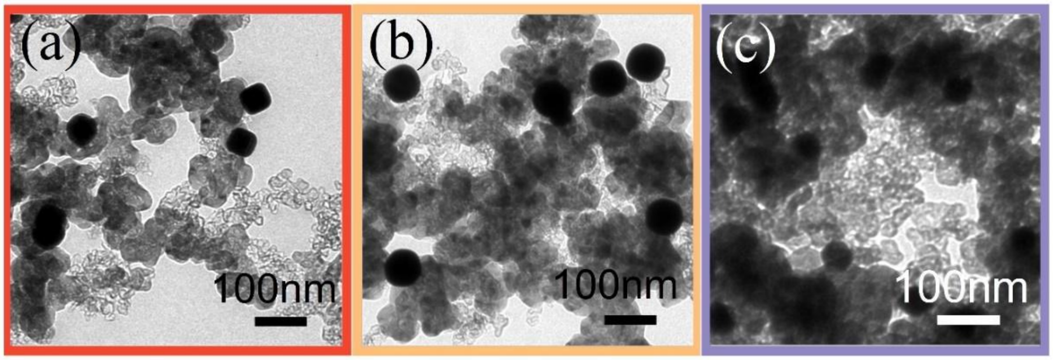


**Figure S3.** TEM images of Au/C composites of (a) Au NCs, (b) Au RDs and (c) Au OCs.

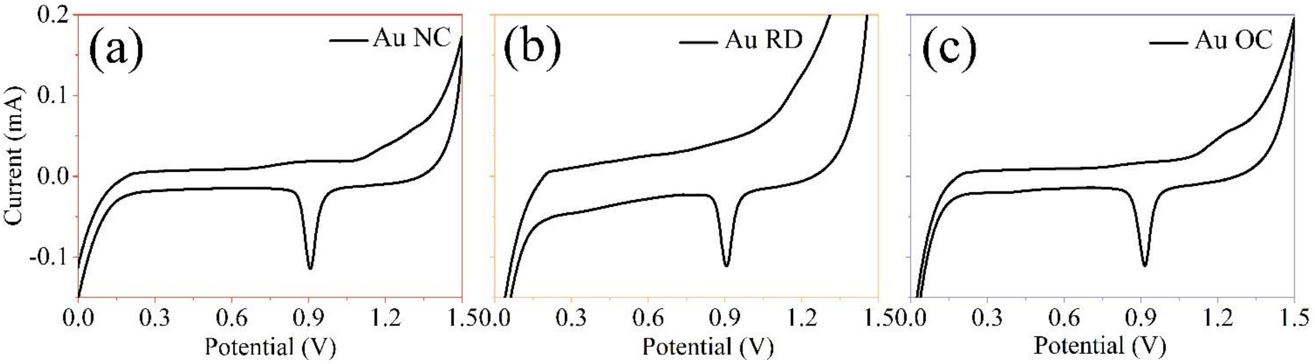


**Figure S4.** CV curves on (a) Au NCs, (b) Au RDs and (c) Au OCs in the non-Faradaic regions of 0 to 1.5V with a scan rate of 10 mV/s in the electrolyte of 0.1 M $HClO_4$ solution. The peak areas of CV curves were integrated and then divided by the scan rate and conversion factor of 450 $\mu C/cm^2$ to get the ECSA of electrodes. The specific ECSA of electrodes are shown in Table S3.

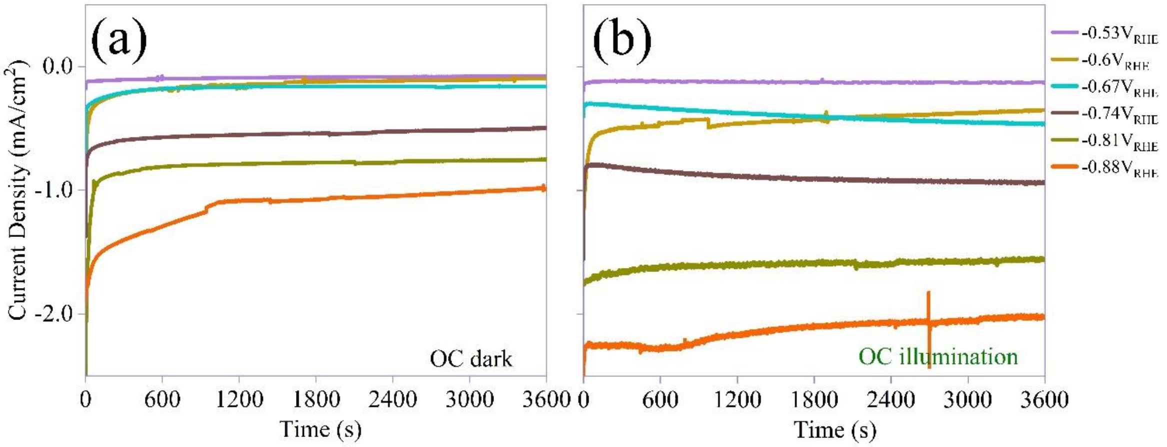


**Figure S5.** Current density on Au NCs in 3600 seconds time in $CO_2$RR system under potentials from -0.53$V_{RHE}$ to -0.88$V_{RHE}$ with a step of 0.07$V_{RHE}$ (a) in dark and (b) with illumination.

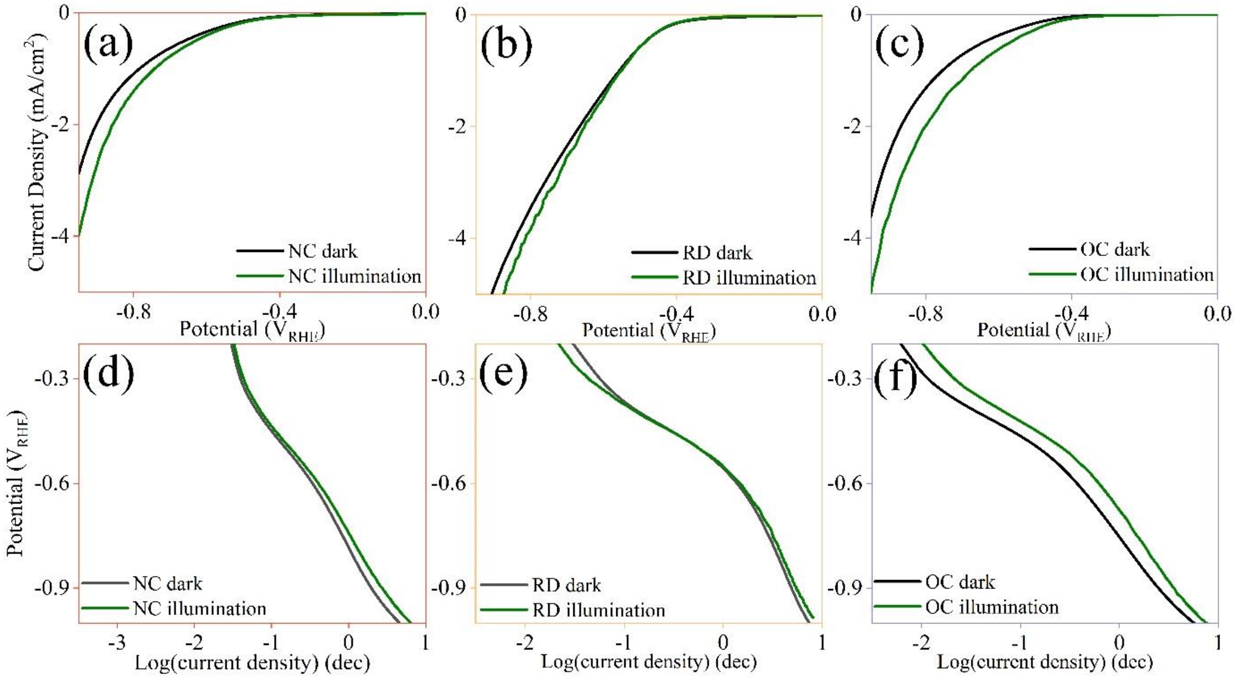


**Figure S6.** LSV curves and Tafel plots of (a, d) Au NCs, (b, e) Au RDs and (c, f) Au OCs in $CO_2RR$ system in dark conditions (black) and with 525 nm LED lightening (green).

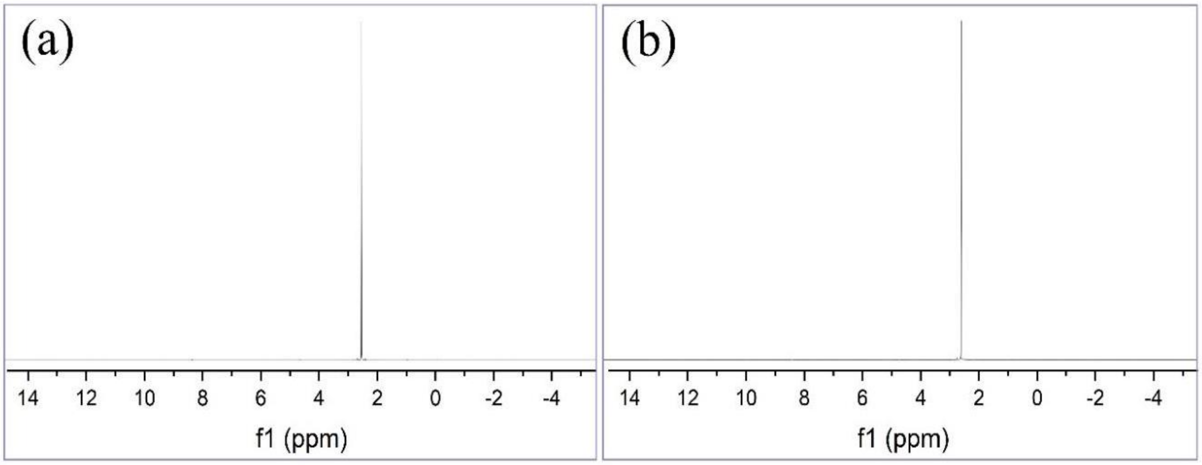


**Figure S7.** $^1$H NMR spectrum of electrolyte after $CO_2$RR measurement on Au OCs (a) in dark conditions, (b) with constant 525 nm LED illumination. In our system, the internal standard DMSO showed its chemical shift at 2.6 ppm in the spectrum. For other Au NPs, the spectrum are similar to OCs. No obvious peak corresponding to liquid products is observed on all three Au NPs in both situations.

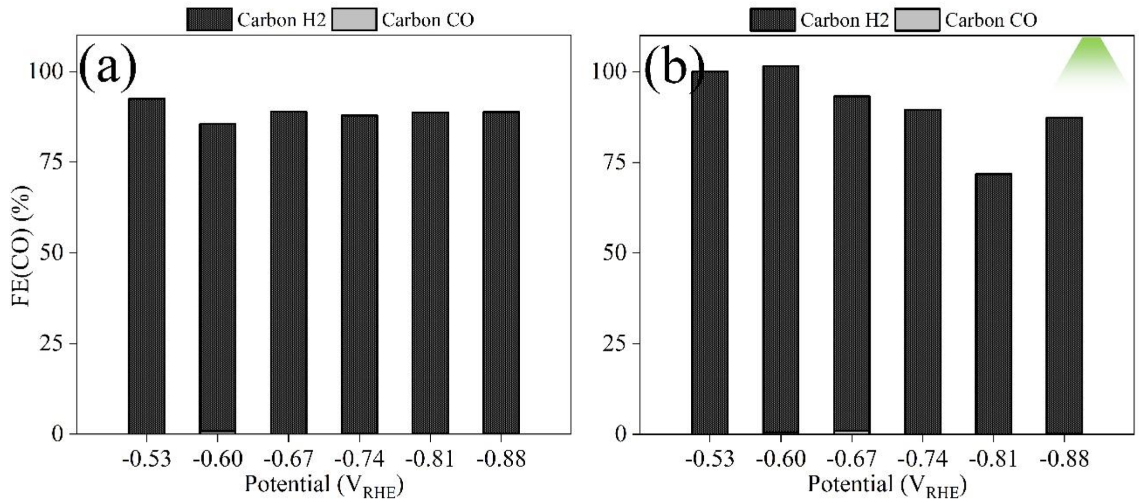


**Figure S8.** Control experiment of $CO_2$RR system based on carbon. FE(CO) and FE($H_2$) (a) in dark condition, (b) with 525 nm illumination.

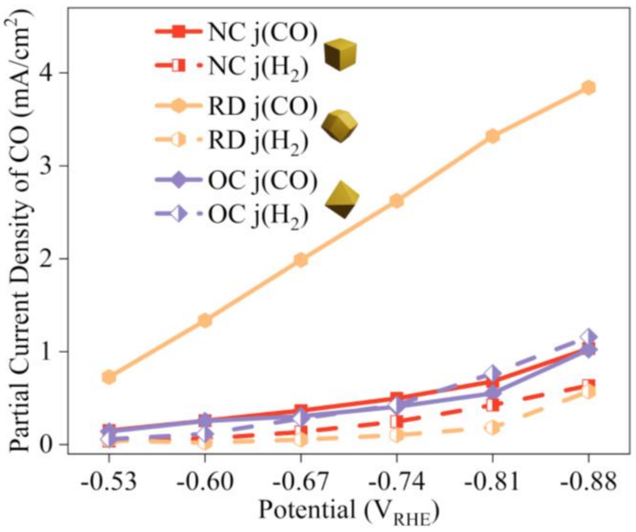


**Figure S9.** Partial current density of CO and $H_2$ on Au NCs, RDs and OCs in electrocatalytic $CO_2RR$ system.

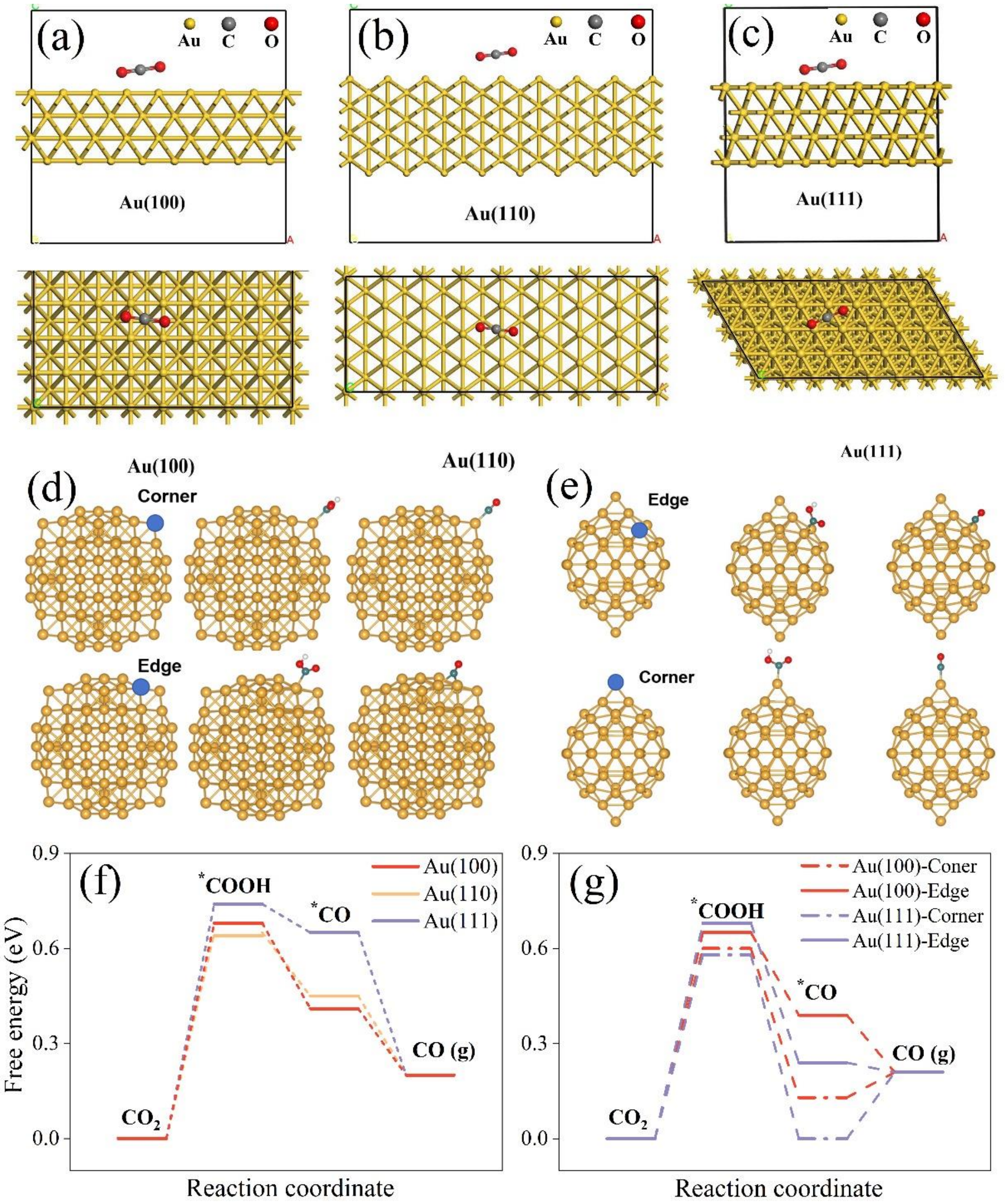


**Figure S10.** Model of DFT calculation: side views (top half) and top views (bottom half) of 4 × 2 periodic surface slab including four atomic layers of (a) Au(100) (b) Au (110) (c) Au (111). Au cluster model for edge and corner free energy calculation with exposed facets as (d) Au (100), (e) Au (111). (f) Calculated free energy diagrams for the $CO_2$ reduction process on Au (100), (110) and (111) facets and (g) free energy diagrams for the $CO_2$ reduction on edges and corners.

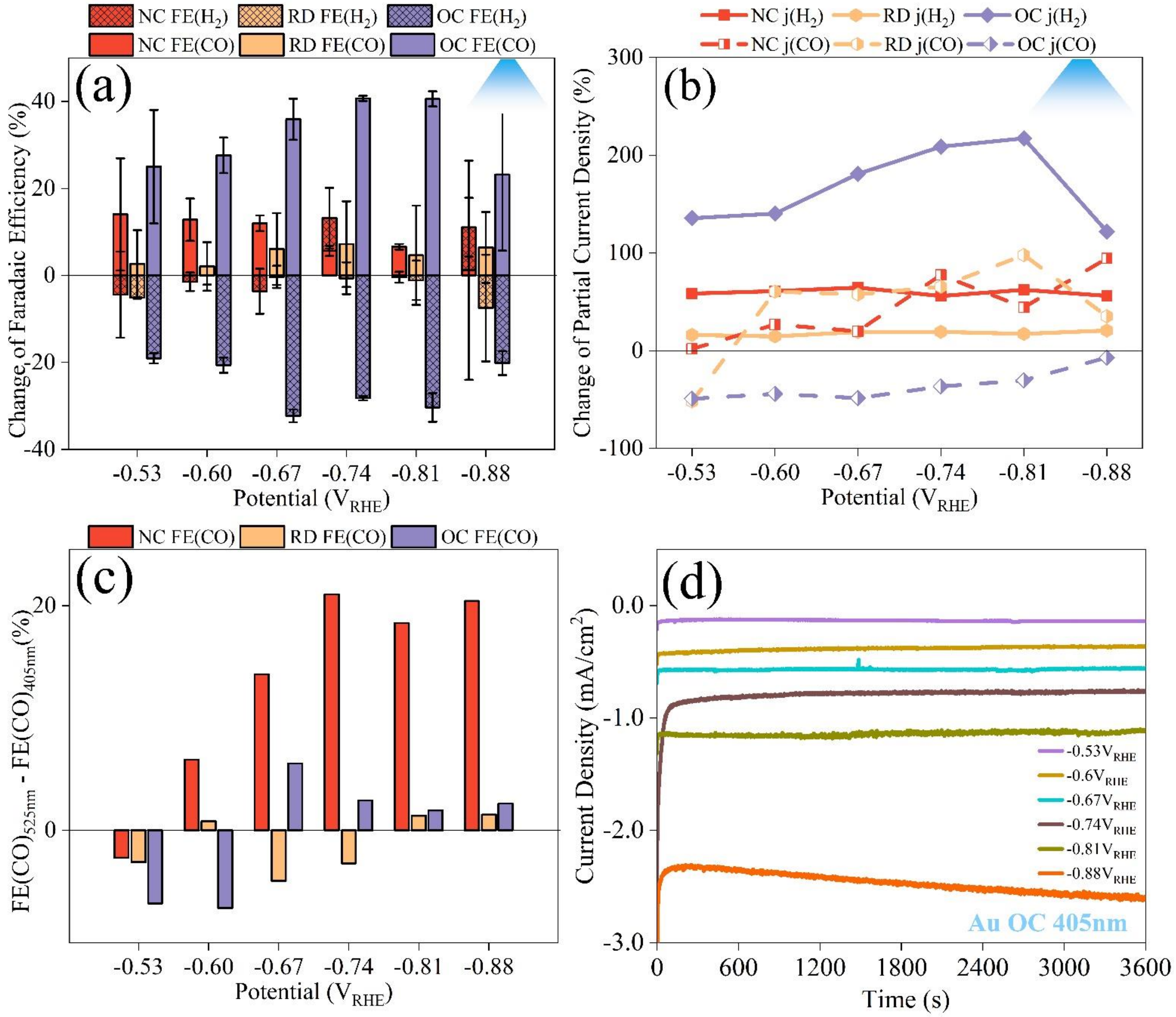


**Figure S11.** $CO_2$RR performance under 405 nm illumination. (a) Change in the absolute value of FE(CO) and FE($H_2$) when illuminated by 405 nm LED compared to dark conditions. The error bars denote the standard deviation obtained from three independent measurements. (b) Percentage change in j(CO) and j($H_2$) when illuminated by 405 nm. (c) The difference between $FE(CO)_{525nm}$ and $FE(CO)_{405nm}$. (d) A typical chronoamperometry measurements under 6 measured potentials used for computing FE and change of current density under 405 nm.

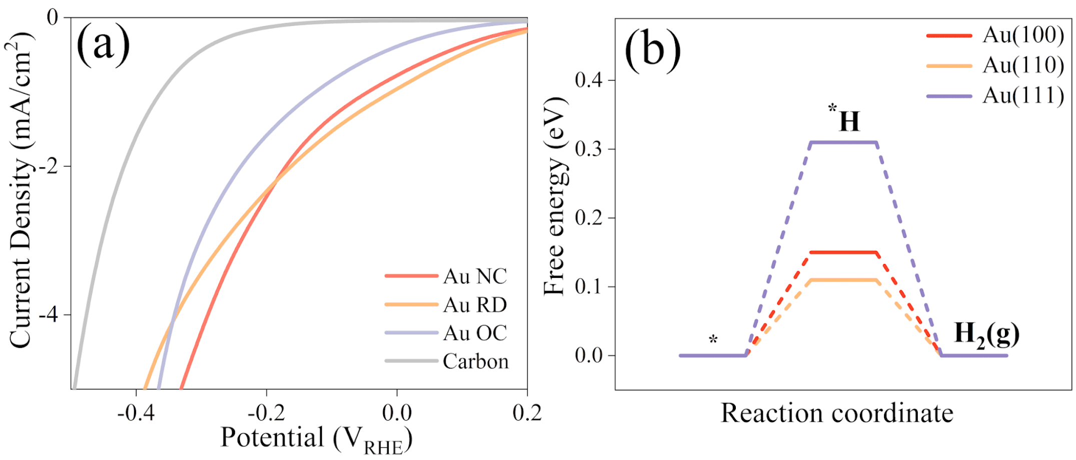


**Figure S12.** HER performance on Au NPs. (a) LSV curves of Au NPs in electrocatalytic system. (b) Calculated free energy diagrams for the HER process on Au {100} {110} and {111} facets.

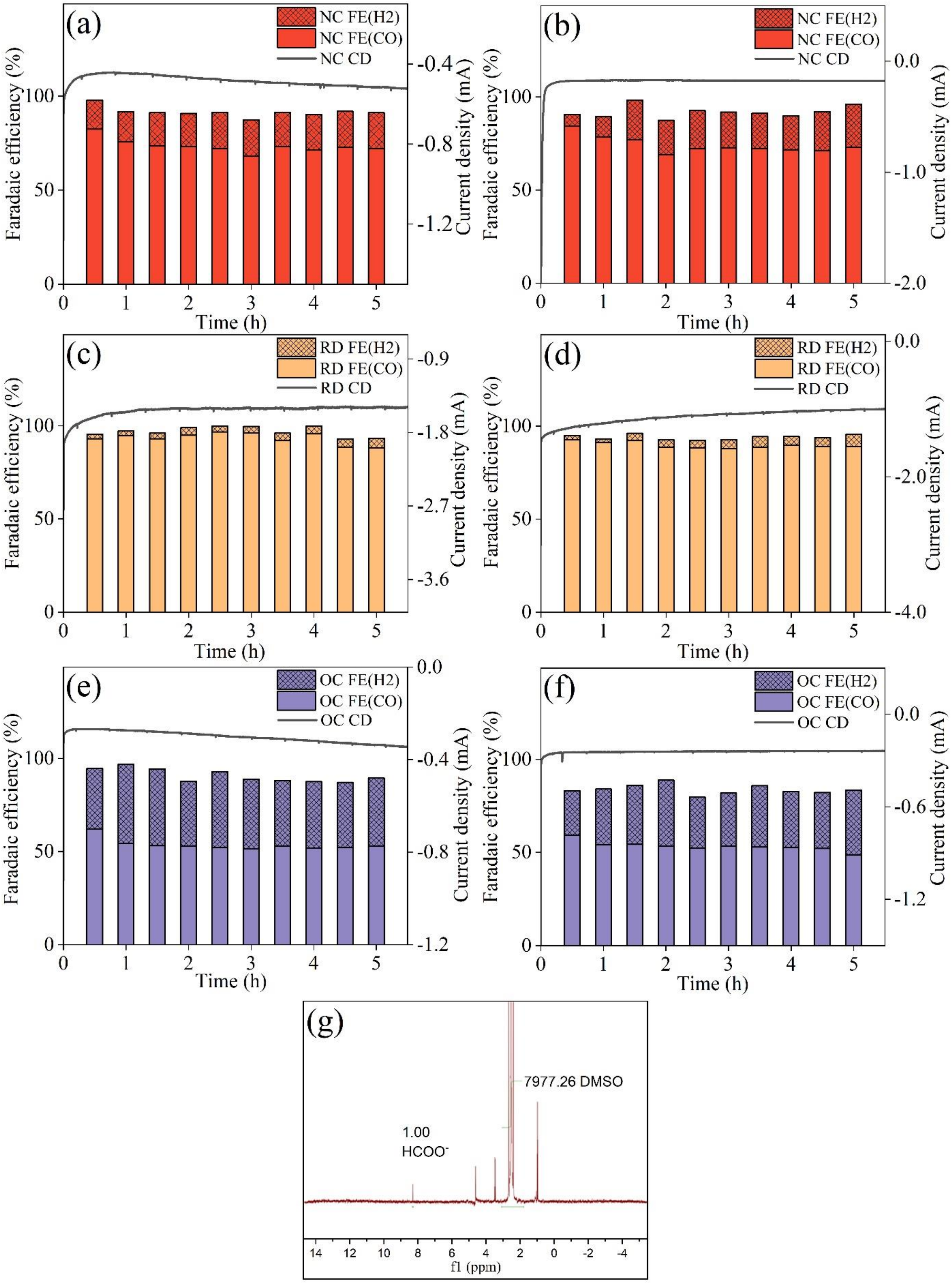

(a)
NC FE(H2)
NC FE(CO)
NC CD
Faradaic efficiency (%)
Current density (mA)
Time (h)
(b)
NC FE(H2)
NC FE(CO)
NC CD
Faradaic efficiency (%)
Current density (mA)
Time (h)
(c)
RD FE(H2)
RD FE(CO)
RD CD
Faradaic efficiency (%)
Current density (mA)
Time (h)
(d)
RD FE(H2)
RD FE(CO)
RD CD
Faradaic efficiency (%)
Current density (mA)
Time (h)
(e)
OC FE(H2)
OC FE(CO)
OC CD
Faradaic efficiency (%)
Current density (mA)
Time (h)
(f)
OC FE(H2)
OC FE(CO)
OC CD
Faradaic efficiency (%)
Current density (mA)
Time (h)
(g)
7977.26 DMSO
1.00
HCOO-
f1 (ppm)

**Figure S13**. Faradaic efficiencies (FE) of CO and $H_2$ production as well as the corresponding current density on Au NCs (red), RDs (yellow) and OCs (purple) in long term (5h) electrocatalytic $CO_2$ reduction system under (a,c,e) -0.67 $V_{RHE}$ and (b,d,f) -0.74$V_{RHE}$. (g) A typical $^1H$ NMR spectrum of the electrolyte after 5h of experiments. For all rounds, only very limited amount of $HCOO^-$ was detected and the corresponding FE were all less than 1%.

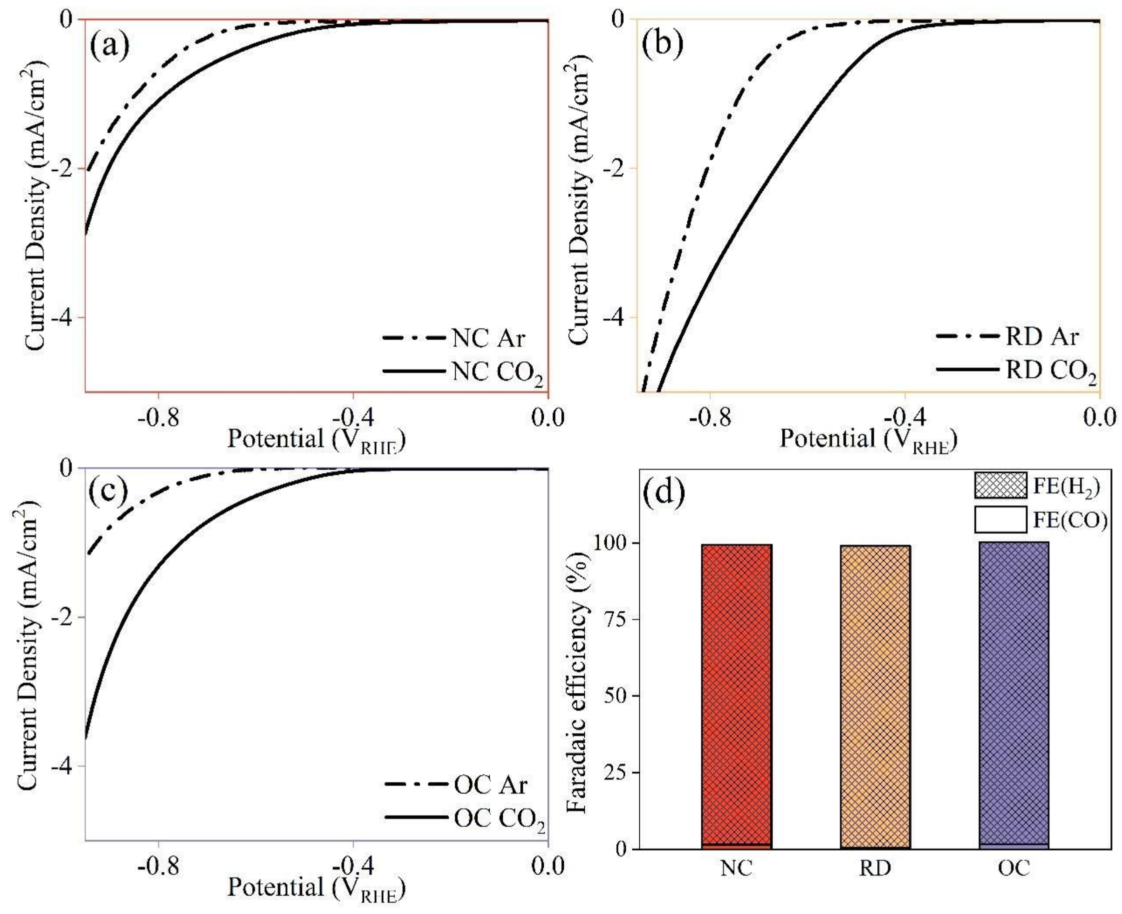


**Figure S14**. Control experiment under Ar bubbling. (a-c) Current density under $CO_2$ and Ar on (a) NCs, (b) RDs and (c) OCs. (d) Corresponding FE of gaseous products.

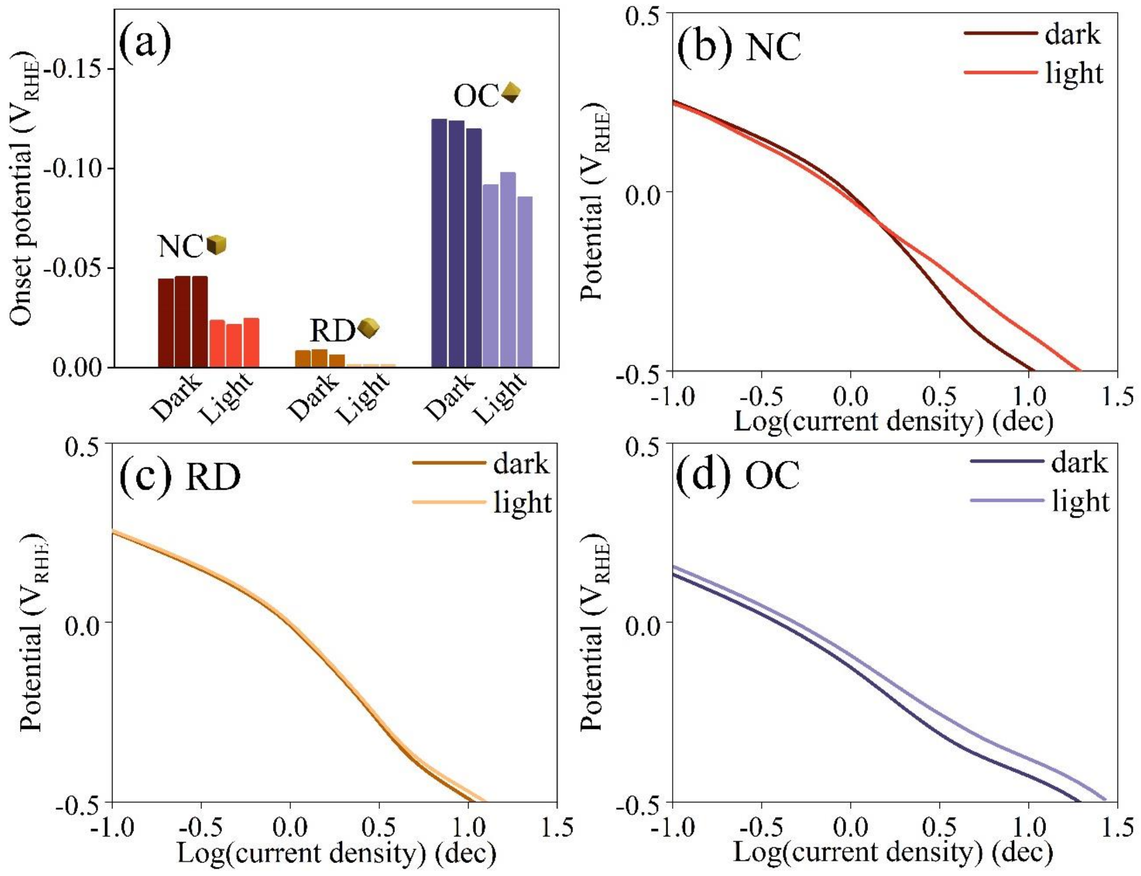


**Figure S15**. HER experiment results. (a) Onset potentials for HER on three Au NPs under dark conditions and 525 nm illumination. (b-d) Tafel plots for (b) Au NCs, (c) RDs and (d) OCs.

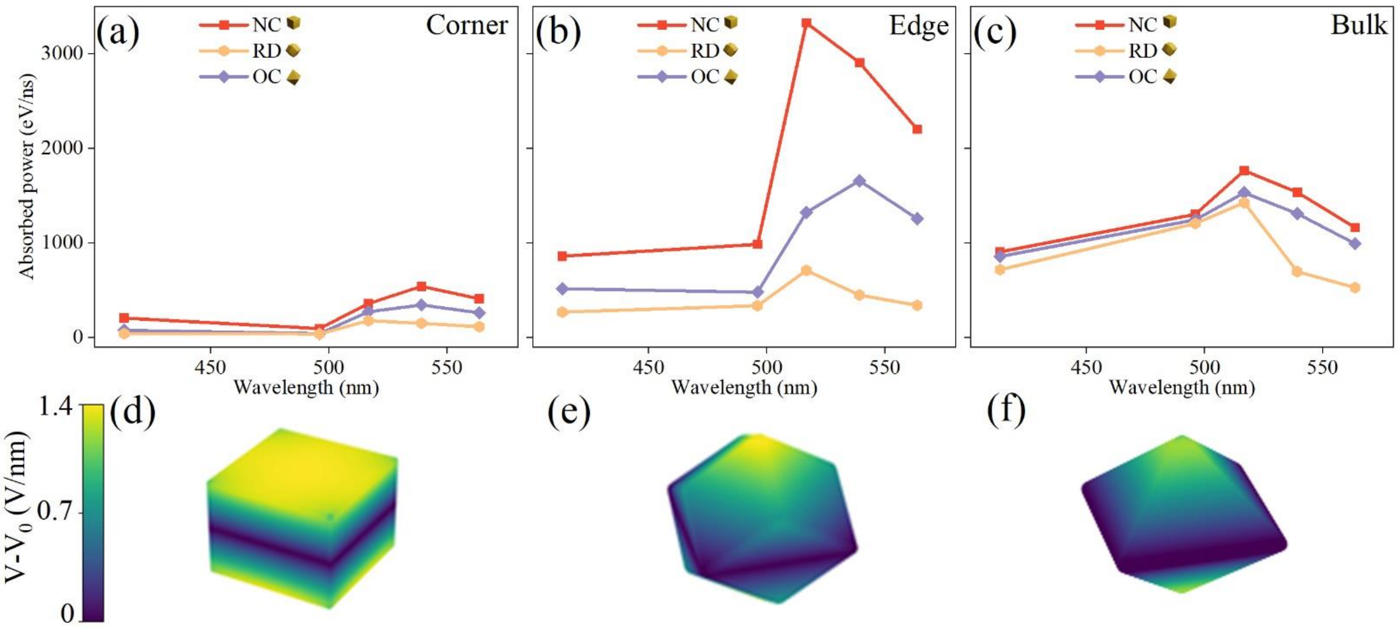


**Figure S16.** Mechanism investigation for the system with 405nm illumination. (a-c) Energy absorption per atom for different region on Au NPs as a function of frequency. (d-f) Electric potential enhancement on Au NPs with 405 nm illumination.

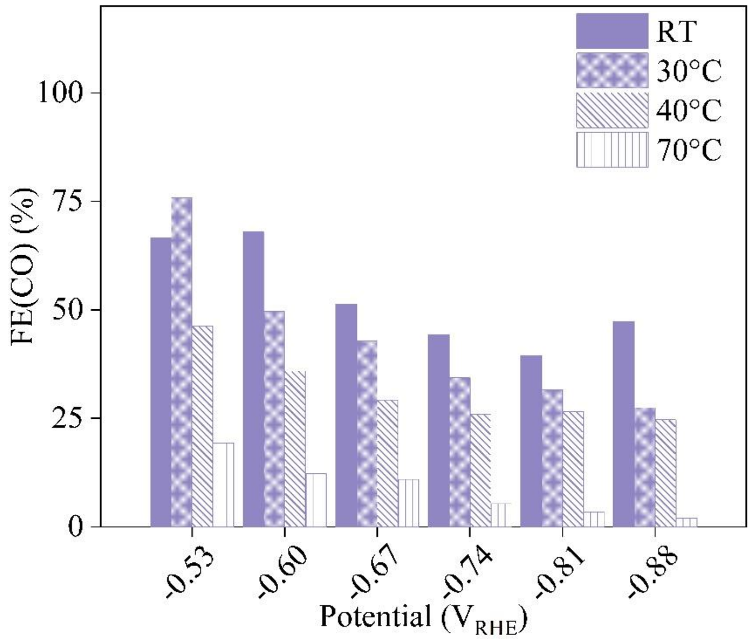


**Figure S17.** FE(CO) on Au OCs under room temperature, 30°C, 40°C and 70°C.

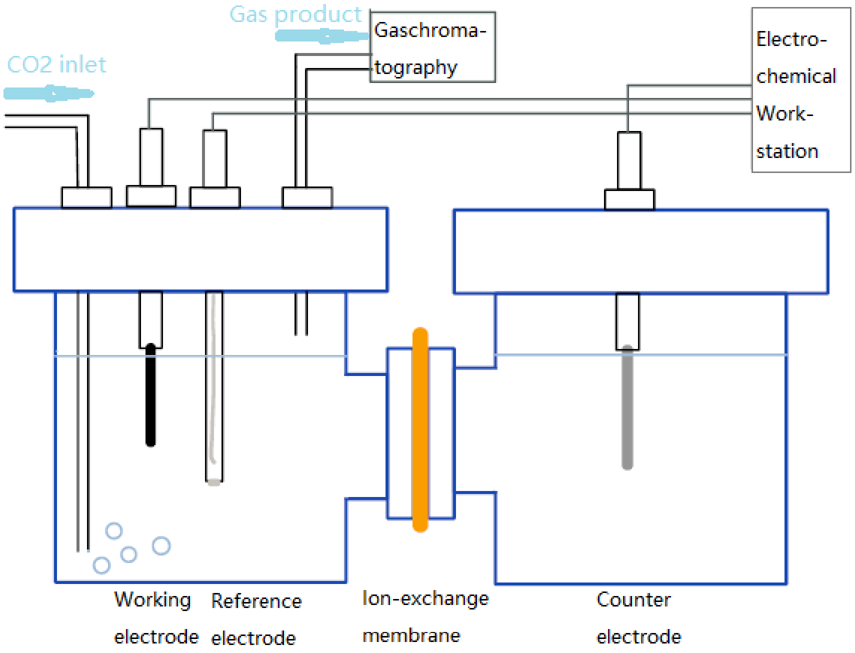


**Figure S18.** Scheme of the setup for $CO_2$RR measurement.

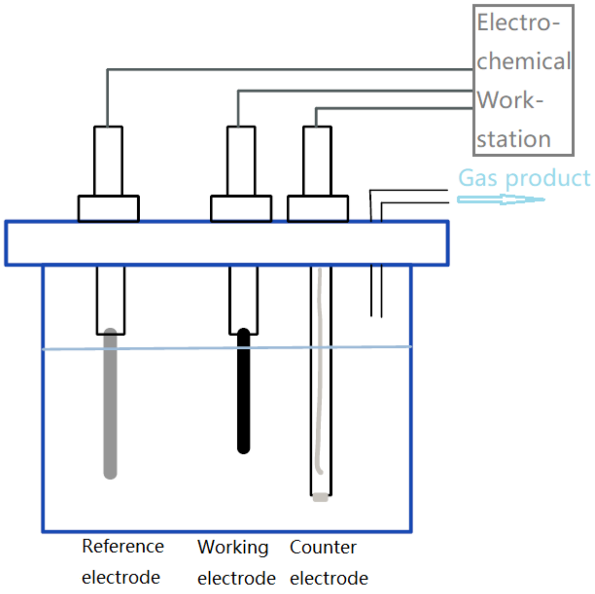


**Figure S19.** Scheme of the setup for HER measurement.

## TABLES

**Table S1.** Expected XRD positions of Au in the FCC phase for Mo Kα radiation.

| 2-Theta(°) | d(Å) | I(f) | (h k l) | Theta(°) | 1/(2d)($Å^{-1}$) | 2pi/d($Å^{-1}$) | n^2 |
|---|---|---|---|---|---|---|---|
| 17.323 | 2.355 | 100 | (1 1 1) | 8.661 | 0.2123 | 2.668 | 3 |
| 20.033 | 2.039 | 52 | (2 0 0) | 10.017 | 0.2452 | 3.0815 | 4 |
| 28.475 | 1.442 | 32 | (2 2 0) | 14.238 | 0.3467 | 4.3573 | 8 |
| 33.516 | 1.23 | 36 | (3 1 1) | 16.758 | 0.4065 | 5.1083 | 11 |
| 35.061 | 1.1774 | 12 | (2 2 2) | 17.531 | 0.4247 | 5.3365 | 12 |
| 40.71 | 1.0196 | 6 | (4 0 0) | 20.355 | 0.4904 | 6.1624 | 16 |
| 44.541 | 0.9358 | 23 | (3 3 1) | 22.271 | 0.5343 | 6.7142 | 19 |
| 45.768 | 0.912 | 22 | (4 2 0) | 22.884 | 0.5482 | 6.8895 | 20 |
| 50.429 | 0.8325 | 23 | (4 2 2) | 25.214 | 0.6006 | 7.5474 | 24 |

**Table S2.** Elemental analysis (C, H, N) test result of Au/C composites.

| Au NP sample | Initial Weight (mg) | Determination CHNS (%) | | | |
|---|---|---|---|---|---|
| | | N | C | H | S |
| Au NCs | 2.044 | 0 | 81.04 | 0 | 0 |
| | 2.298 | 0 | 81.04 | 0 | 0 |
| Au RDs | 2.21 | 0 | 80.75 | 0 | 0 |
| | 2589 | 0 | 80.74 | 0 | 0 |
| Au OCs | 2.078 | 0 | 80.67 | 0 | 0 |
| | 2.587 | 0 | 80.64 | 0 | 0 |

**Table S3.** Electrochemically active surface area (ECSA) of working electrodes.

| Au NPs on electrodes | NC | RD | OC |
|---|---|---|---|
| ECSA ($cm^2$) | 1.28 | 1.11 | 1.28 |

**Table S4.** Edge/facet ratio of Au NCs and OCs.

| Au NPs on electrodes | 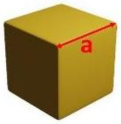  NC | 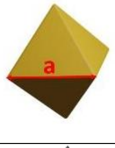  OC | 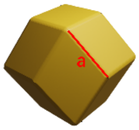  RD |
|---|---|---|---|
| Flat expansion view | 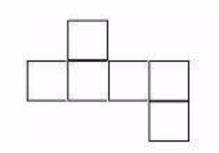 | 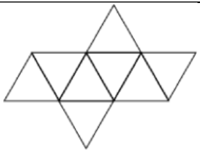 | 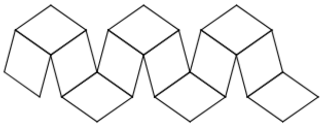 |
| Edge length $a$ /nm | 42.4 | 39.6 | 40.0 |
| Area of edge | $24 \times a \times r$ | $24 \times a \times r$ | $74 \times a \times r$ |
| Area of facet | $6 \times a^2$ | $2\sqrt{3} \times a^2$ | $8\sqrt{2} \times a^2$ |
| Edge/facet ratio | $1.36\times10^{-2}$ | $2.52\times10^{-2}$ | $2.35\times10^{-2}$ |

*Here we assume the edge length is a, the Au atom radius in FCC phase is r, the value of r is 0.144nm. The edge lengths were measured from TEM images.

**Table S5.** Population of hot carriers of intra- and interband transitions.

| Au NP sample | Population of hot carriers in intra- and interband transition under various wavelengths | | | | | | | |
|---|---|---|---|---|---|---|---|---|
| | 563nm | | 539nm | | 516nm | | 496nm | |
| | inter | intra | inter | intra | inter | intra | inter | intra |
| Au NCs | 68% | 32% | 80% | 20% | 85% | 15% | 90% | 10% |
| Au RDs | 85% | 15% | 85% | 15% | 91% | 9% | 94% | 6% |
| Au OCs | 83% | 17% | 85% | 15% | 89% | 11% | 93% | 7% |
